# Science-wide mapping of institutions based on affiliated authors' impact and research integrity proxies

**DOI:** 10.1098/rsos.260870

**Published:** 2026-10-14

**Authors:** John Ioannidis, Jeroen Baas, Roy Boverhof, Cyril Voyant

**Affiliations:** ^1^ Meta-Research Innovation Center at Stanford (METRICS), Stanford University, Stanford, CA, USA; ^2^ Stanford Prevention Research Center, Department of Medicine, Department of Epidemiology and Population Health, Department of Biomedical Data Science, Stanford University, Stanford, CA, USA; ^3^ Elsevier BV, Amsterdam, The Netherlands; ^4^ Research Intelligence, Elsevier BV, Amsterdam, The Netherlands; ^5^ Centre Observation, Impacts, Energy (OIE), Mines Paris—PSL, Campus Pierre Laffitte, F-06904 Sophia Antipolis Cedex, France

**Keywords:** citations, scientometrics, retractions, institutions, research assessment, self-citations

## Abstract

We aimed to generate institution-level data of indicators that may reflect, as proxies, the impact and the research integrity of the authors affiliated with them and that also adjust for institution size and scientific subfields where these authors work. We used the Scopus database to generate institution-level data on top-cited authors (according to a composite citation indicator) and three indicators that may reflect problems with research integrity: the total number of authorships in papers retracted for reasons other than publisher/journal errors, and subfield-adjusted proportions of top-cited authors who have high (>95% percentile) self-citation rates or high (>95% percentile) rates of publications in Scopus-discontinued titles. There was modest overlap among top institutions based on volume and based on number of top-cited authors. However, there was minimal overlap when institutions with the highest proportions of top-cited authors were considered; these included mostly research institutes and tech organizations and only a minority of universities. Institutions with the highest self-citation, discontinuation and retraction penalties clustered in specific countries such as Saudi Arabia, People's Republic of China, Malaysia, Iran, India and Indonesia. The publicly available datasets that we provide allow the exploration of institution-level research assessments that balance high impact against proxies of diverse aspects of research integrity.

## 1. Introduction

Evaluations of institutions are widely popular and result in diverse appraisals and rankings that often attract justifiable criticism as to their reliability and face validity [1–4]. Most evaluations typically try to measure impact using different indicators of excellence, influence, reputation and recognition. In most of these assessments, there is very limited or no attention to issues of research integrity. For example, rankings that focus on impact typically do not account for all the behaviours and errors that may lead to retractions. They are also typically entirely silent to demands that may lead some scientists to high publication and citation counts through spurious practices. Another fundamental problem with such assessments is that information on how many people are employed by a university or other institution is difficult to collect in a standardized way across institutions worldwide. There are many different types of faculty and staff, including tenured and non-tenured, different ranks, regular and adjunct appointments, as well as hierarchies in universities versus other organizations that publish research. The exact categories and which ones are counted by each institution may vary a lot. This heterogeneity generates biases when comparing different institutions directly and when normalizing impact metrics by the number of affiliated researchers.

Several assessment systems try to capture the number of people affiliated with each institution that have achieved some specific hallmark of impact, e.g. Nobel prizes or other major recognitions. However, for the most globally acknowledged—and thus by definition rare—recognitions, only few institutions have any such individuals. Many assessment systems end up using numbers of published papers, citations or numbers and proportion of papers in percentiles of citations. Absolute numbers of papers and citations, however, would again require some adjustment for the number of available producing authors to be more meaningfully interpretable. Proportion of papers in citation percentiles does achieve some reasonable adjustment [5] but provides no information on institutional size and on whether the high-impact work is produced by a few individuals or spread across many. Moreover, diverse research integrity issues remain completely ignored and unaccounted for.

Here, we use the Scopus database [6] and previously derived datasets of scientific subfield-adjusted lists of top-cited scientists [7–10] to derive standardized, institution-level metrics that may be used for appraising the volume and impact of diverse institutions across science and around the world. For each institutional affiliation, we leverage these resources to generate information on the overall number of publishing authors and the number of publishing authors who fulfil pre-defined threshold criteria for onset of their publication age, their overall publication volume and their overall volume of publications as single, first or last authors. Instead of selecting institution-specific categories of employees, we count all authors with an institutional affiliation and separately specifically those who have started publishing in the last 45 years (a typical duration of a scientific career) and who have demonstrated substantial productivity. Moreover, for each institution, we obtain the number of authors who are top-cited in their scientific subfield overall and after using the same filtering thresholds. The proportion of top-cited authors among all authors with such filtering thresholds may then be considered as a first adjusted benchmark of impact for each institution. Finally, we propose that impact should not be examined alone but seen in combination with indicators that may be proxies of diverse aspects of research integrity. Therefore, we add further adjustments that consider for each institution the proportion of its eligible top-cited authors who have high self-citations and high share of their papers published in journal titles that have been discontinued for quality concerns by Scopus; and, furthermore, the total authorships of retracted papers not owing to publisher/journal error. These adjustments aim to balance the citation impact against indicators that may reflect, as proxies, diverse aspects of research integrity. Strengths and caveats of such an approach to assessing institutions are then discussed and put into perspective.

## 2. Methods

### 2.1. Scopus database

We used the Scopus database [6] with a data freeze on 1 August 2025 [11]. Similar to previous work [7–10], we limited all analyses to authors who have published at least five full papers in the database (defined as articles, reviews and conference papers in Scopus), yielding a total of 10 933 183 eligible authors. The accuracy of Scopus for assigning all papers to the correct author and not splitting papers of the same author to different author files is deemed very high [6] including also for authors of Asian names that were more problematic to disentangle in earlier years. A study found precision and recall of 99% and 98%, respectively, for Japanese researchers [12], while an average precision of 98.1% and an average recall of 94.4% were reported in a 2020 internal overview of the database [6].

### 2.2. Aggregation of organizations

The merging of author affiliations to ‘aggregated’ organizations (which we refer to as *institutions*) is done according to the Scopus process for assigning one primary affiliation to each author and for creating hierarchies of organizations that are mapped to each specific institution. Details are discussed in Appendix Methods in the supplementary material. The full hierarchy of organizations mapped to each institution can be found in the subscription edition of Scopus.

### 2.3. Top-cited authors

For each organization and institution, the number of top-cited authors is counted according to the latest edition (August 2025) of the citation indicators databases [11] that include all authors who are in the top 2% of their scientific subfield (across 174 subfields classified by Science-Matrix [13] or among the top 100 000 of all science based on a composite citation indicator. The composite citation indicator combines six citation indicators, including total citations, Hirsch h-index, Schreiber co-authorship-adjusted hm-index, citations to single-authored papers, citations to single- or first-authored papers and citations to single-, first- or last-authored papers [7–10]. It aims to accommodate co-authorship and indirect measures of author contributions through author positions since reliable, detailed and standardized data on author contributions are not available across the scientific literature. Some fields use alphabetic listing, in which case first and last author positions do not carry particular importance; however, we have previously estimated that alphabetic listing may affect only a small minority of papers [8]. Authors are considered in the top-cited list if they manage to pass the selection thresholds in analyses excluding self-citations or in analyses including self-citations. Moreover, separate datasets of top-cited authors are generated for career-long citation impact and for citation impact limited to the citations received in the single most recent year (here, this is calendar year 2024). Of the 10 933 183 authors with at least five full papers in Scopus, a total of 230 333 and 236 313 are captured as top-cited in the career-long and single recent-year datasets, respectively. In the Results, we first focus on career-long data, since they reflect cumulative long-term influence. The single recent-year analyses may be more vulnerable to gaming, e.g. through fake papers and orchestrated self-citation cartels, and they are presented separately and briefly compared with career-long results.

### 2.4. Threshold filters for authors and proportions of top-cited authors

We have derived data for the number of authors and the respective number of those who are included in the datasets of top-cited authors using threshold filters for publication age and for productivity volume and patterns.

#### 2.4.1. Publication age

For publication age, we used a filter for starting to publish (any item that is Scopus-indexed) in 1980 or later. This allows for up to 45 years of publication history, excluding authors who started publishing in earlier years. Assuming one publishes their first paper usually in their 20s to early-30s, a 45-year window covers the time to typical retirement for a full academic or other research career. For any future updated versions of the ranking, the 45-year window may be a rolling time window. We also derived data for sensitivity analyses where we further excluded authors who have not had any Scopus-indexed items published from 2020 onwards. These sensitivity analyses further exclude authors who may be dead or may be scientifically inactive.

#### 2.4.2. Productivity volume

For productivity threshold filters, we used one filter based on the number of total Scopus-indexed published items and another one for single-, first- or last-authored items. For the number of total published items, we derived data for the number of authors with 40 or more total published items. Authors with <40 published items have negligible chances of being inducted in the top-cited list (97% of the top-cited authors have 40 or more published items). For the filter of single-, first- or last-authored items, we set the filter at having five or more such publications; 99.7% of the top-cited authors have five or more such publications.

We derived the proportions of authors who are included in the datasets of top-cited authors for career-long impact and separately for single recent-year impact. When all authors are considered without further filtering for publication age, many included authors may represent individuals that reflect the long past legacy of the institution; this may affect, to a different extent, institutions with older versus only recent history. When no filter for the number of total published items is set, institutions who are more actively engaging many early career and in-training researchers may have a large denominator of total eligible authors than those that have few such researchers and a larger share of well established, senior scientists. Early career and in-training researchers have minimal chance of reaching top-cited status so fast [14], therefore the proportion of top-cited authors would penalize institutions with many such researchers unless they are filtered out in the calculations. Finally, when no filter is set for a minimum number of single-, first- or last-authored papers, authors who have not much of a trajectory of leading roles in research may also distort the percentage of top-cited authors, since these authors rarely make it to the lists of top-cited authors based on the composite citation indicator used. As above, the composite citation indicator placed major emphasis on single-, first- or last-authored contributions.

In the Results section, we present first data on all authors with at least five Scopus-indexed full papers and then we focus primarily on the subset of those who started publishing in 1980 or later, have published 40 or more Scopus-indexed items in their career, and these include at least five single-, first- or last-authored items. The selected subset for this analysis (henceforth called *primary analysis*), may approximate the group of senior active faculty in universities or equivalent leading roles in other institutions and may be most appropriate for standardized institutional comparisons rather than the all-inclusive authors' data.

### 2.5. Analysed institutions

A very large number of organizations may have very few authors assigned to them even at the aggregated institutional level. Any evaluation of the number or proportion of highly cited authors in them would not be meaningful given the sparse information. Therefore, we first generated data focused on only those institutions that included at least 50 authors who have had at least 15 Scopus-indexed items. Authors with 15 publications can be considered to have a well-established publishing career as opposed to those with lesser, more sporadic output. These data are made available in public repositories for further exploration by interested users. However, we realized that this filter includes many institutions with too few authors meeting the primary analysis criteria. Therefore, we finally generated percentile rankings focused exclusively on institutions that have at least 100 authors eligible by the primary analysis filters.

Scopus uses the latest listed affiliation in the most recently published items in assigning where an author is even if the author may have migrated across different institutions in the past. For each institution, its country is also allocated as per Scopus. Universities can usually be assigned unambiguously, while some companies may be multinational and have employees in many countries.

### 2.6. Indicators that may be proxies of diverse aspects of research integrity

The research integrity of authors and their institutions can be evaluated with diverse indicators that capture different, potentially complementary aspects. These indicators function as proxies rather than firm proof of research misconduct or gaming practices. Each of them may offer some insights into research practice and malpractice and consideration of multiple indicators may offer a more comprehensive view rather than focusing on a single one. Here, we used three indicators that capture features that are previously recognized to reflect directly or indirectly problems in research integrity and where there is access to science-wide data: exceedingly high rates of self-citations, publications in titles that have been discontinued owing to quality concerns and retractions owing to author issues (rather than publisher/journal error). These indicators had previously been brought together in the Research Integrity Risk Index (RI²), a framework introduced in 2025 that combines institutional research integrity indicators into a composite score and corresponding risk tiers [15]. Moreover, science-wide data is available for these indicators, as described below. Of note, we use the term ‘research integrity’ to include both research and its publishing process in the wider sense and there may be substantial heterogeneity in the elements affected. For example, retracted papers may be seriously flawed in their methods or their data, or may exhibit diverse violations of publication ethics. Sometimes the retracted papers may even be totally sound scientifically but may be retracted because of excessive text duplication or gift authorship. Similarly, delisting journals from Scopus may reflect concerns for very diverse types of manuscript handling in these journals, but it does not mean that all journal articles published in these journals contain sketchy, unethical or wrong research.

We have previously linked retraction data for each author in the Retraction Watch database [16] with the Scopus-based author citation datasets with [17,18]. As described previously, we consider only retractions not owing to publisher/journal errors and these represent the majority of catalogued retractions [16–18]. Top-cited authors are few for most institutions and authors with retractions may not reach or may fall off the range of being top-cited. Therefore, we consider all institutional authors who started publishing in 1980 or later and had at least five published items, we count for each the number of their publications not owing to publisher/journal error and then sum all these retraction authorships, Σ*R*. Illustratively, a single retracted paper with three institutional authors contributes three retraction authorships for that institution.

Self-citations may often be fully appropriate to put new work in the context of older work. Moreover, authors in earlier career stages or in extremely niche fields may appropriately have higher self-citation rates. Therefore, we focused only on top-cited authors. Since it is impossible to evaluate in depth whether each self-citation is appropriate, for each top-cited author we note whether their percentage of self-citations is higher than the threshold of the 95% percentile for self-citations in the respective subfield among the top-cited author peers. We already generate such data on self-citations and subfield thresholds (considering self-citations from all authors of a given paper) in the annually updated citation datasets (and high rates of self-citations [10,19] and here we used the August 2025 edition [11].

In a similar fashion, for each top-cited author we note whether their percentage of published items that appeared in titles that have been discontinued by Scopus is higher than the 95% percentile for items in discontinued titles among the top-cited author peers in the respective subfield. Discontinuation of a title here means that after a title is selected for re-evaluation and flagged for discontinuation by the Content Selection and Advisory Board (CSAB) [6], new content will no longer be included in Scopus. Discontinuation means that the title no longer meets the quality standards set by CSAB. For details on how quality is appraised see [20]. Already indexed content from this title will remain in Scopus unless severe unethical publication practices can be proven, in which case indexed content may be removed (a very rare occurrence). Journals that simply cease to publish are not considered ‘discontinued’; the term is reserved for active decisions by the CSAB.

### 2.7. Summary score considering both impact and research integrity proxy indicators

For each institution, we generate a score that combines information on both the citation impact and research integrity proxy indicators. This score is given by

$$\begin{array}{c} \frac{N\left(t\right)-\left[\frac{\Sigma R}{2}+\left(S\left(t\right)-\left(0.05\right)N\left(t\right)\right)+\left(D\left(t\right)-\left(0.05\right)N\left(t\right)\right)\right]}{N\left(a\right)}, \end{array}$$

where *N(t)* is the number of top-cited authors in the primary analysis, *N(a)* the number of all authors in the primary analysis, *S(t)* the number of top-cited authors in the primary analysis with self-citations above the 95% percentile, *D(t)* the number of top-cited authors in the primary analysis with percentage of items published in discontinued titles exceeding the 95% percentile and Σ*R* the sum of all retraction authorships across all institutional authors with at least five published items who started publishing in 1980 or later.

In this framework, retractions by any author always penalize an institution and the loss owing to having two retracted authorships is equivalent to the gain from having a top-cited author. Conversely, the proportion of its top-cited authors with high self-citations and high rates of publications in discontinued titles may offer a bonus or penalty to the institution depending on whether they are less or more than 5%. Our weighing of retraction authorships and use of 5% baseline rates for high self-citations and discontinued-title publishing have a largely arbitrary nature. We have chosen to offset one top-cited scientist by two retraction authorships which may be seen as a high penalty. However, we worry that citation metrics and rankings (including the ones we have produced and annually update) are used indiscriminately and excessively without any attention to ‘negative’ metrics, such as retractions. Therefore, we make here an effort to draw more attention to such a negative metric with the hope that this may incentivize more rigorous standards of research and greater attention to retractions. As for the best percentage threshold of self-citations, this cannot be too low, since then most authors who are tagged as having high self-citations will not be abnormally excessively self-citing, or too high, since then most abnormal excessive self-citers will be missed. For similar reasons (not too low, not too high), we have chosen a 5% discontinued-title publishing threshold.

Of note, retraction authorships are counted for all eligible authors in an institution, while the other two proxy indicators focus on the eligible top-cited authors only. The reason for this choice is that authors with retracted work not owing to publisher/journal error may suffer a considerable citation penalty [21,22] that keeps them away or removes them from the top-cited list, while there is no evidence for such a citation penalty for those with high values on the other two proxy indicators. Also of note, we assign the same retraction penalty to each co-author of a retracted paper and thus to their institutions, even though it is possible that different authors and different institutions within a retracted paper may have had different levels of responsibility for the aspects that led to the retraction. However, for the vast majority of retracted papers, there is no way to assign responsibility to specific authors and specific institutions among the several/many listed in the author masthead.

We should also acknowledge that for journal titles that were discontinued by Scopus, their quality may have been suboptimal since their inception, for a long while or only relatively recently before their discontinuation. There is no information that would allow us to arbitrate when their quality suffered, therefore we cannot isolate only papers published during a time period of suboptimal standards.

### 2.8. Percentile ranking of institutions

These summary scores were used to generate institutional percentile rankings. We also performed extensive sensitivity analyses of the institutional percentile ranking by varying the main tuning parameters of the metric. Specifically, we changed the self-citation and discontinued-titles threshold percentiles (*p* = 80, 95, 99) and divided the contribution of retraction authorships Σ*R* by factors *f* = 1, 2 (baseline) and 4. For each combination of (*p, f*), we recomputed the adjusted score for institutional percentile ranking using

$$\begin{array}{r} \frac{\left\{N\left(t\right)-\left[\frac{\Sigma R}{f}+\left(S\left(t,p\right)-\left(1-p/100\right)N\left(t\right)\right)+\left(D\left(t,p\right)-\left(1-p/100\right)N\left(t\right)\right)\right]\right\}}{N\left(a\right)}, \end{array}$$

where *N(t)* is the number of top-cited authors in the primary analysis, Σ*R* is the number of retraction authorships among eligible authors, *S(t,p)* is the number of top-cited authors whose self-citation percentage exceeds the *p*-th percentile in their subfield and *D(t,p)* is the number whose share of publications in discontinued titles exceeds the subfield-specific *p*-th percentile.

We then quantified agreement with the baseline (*p* = 95, *f* = 2) ranking using Spearman rank correlations for both the institutional percentile ranks and the adjusted percentage of top-cited authors. To visualize potential shifts, we generated scatter plots and Bland-Altman plots of the baseline versus sensitivity-analysis rankings and adjusted percentages. We also estimated smoothed distributions using kernel density estimation to visualize the distribution of adjusted institutional scores across specifications for each choice of (*p, f*).

## 3. Results

### 3.1. Overall data with institutions

Data are provided in the publicly accessible link on a total of 6979 institutions with ≥50 authors who have ≥15 Scopus-indexed items. These institutions cover 78% of all authors with at least five full publications (8 524 471/10 933 183) and the vast majority of career-long top-cited authors (90.0% [207 256/230 333]). When limited to authors eligible for the primary analysis (those who started publishing in 1980 or later, have published 40 or more Scopus-indexed items in their career, and these include at least five single, first- or last-authored items), there were 1 214 702 authors across these 6979 institutions, of which 145 704 (12%) were top-cited according to career-long impact. 22 823 authors (2781 top-cited ones) were counted for more than one aggregated affiliation.

Of the 6979 institutions, 2380 have also at least 100 authors who started publishing in 1980 or later, have published 40 or more Scopus-indexed items in their career, and these include at least five single-, first- or last-authored items and are thus used for presented percentile rankings.

### 3.2. Institutions with highest numbers of top-cited authors

Table S1 in the supplementary material and table 1 present information on the 100 institutions that have the highest number of top-cited authors in the primary analysis (range 278–1135 top-cited authors per institution). The listed institutions are highly recognizable organizations with undisputable major presence in science. However, they vary a lot on the proportion of top-cited authors in the primary analysis from 6.98% (Shanghai Jiao Tong University) to 40.13% (University of California, Berkeley). Table S1 in the supplementary material also shows, for comparison, data for these institutions considering all authors. There is substantial variation in the share of authors eligible for the primary analysis (range 9–26%) with the lowest values at UC Berkeley and MIT. Variation is even greater in the proportion of top-cited authors who meet the primary-analysis criteria (range 46–99%). Chinese institutions and to a lesser degree also institutions in Singapore and Republic of Korea have almost all their top-cited authors qualify for the primary analysis. This is because in contrast to American, European and Australian institutions, Asian institutions have seen massive growth more recently and thus very few of their top-cited authors are excluded because of old (pre-1980) start of publication history. Nevertheless, Chinese institutions still display the lowest proportion of top-cited scientists within table S1 in the supplementary material and table 1.

**Table 1.** Number of top-cited authors (career-long citation impact) in the 100 institutions with the highest number of top-cited authors in the primary analysis. Data per region, with details shown on top 20 institutions. Detailed data for all 100 institutions appear in table S1 in the supplementary material.

region	number among top 100	among top 20
USA	47	Harvard University (1135), University of Michigan, Ann Arbor (913), Stanford University (912), Johns Hopkins University (782), University of Pennsylvania (781), University of Washington (751), University of California, Los Angeles (687), Columbia University (633), University of California, San Diego (629), Mayo Clinic (592), Duke University (590), Massachusetts Institute of Technology (564), University of Minnesota Twin Cities (551), Cornell University (544)
UK	12	University of Oxford (828), University College London (810), University of Cambridge (651), Imperial College London (542)
France	7	none
People's Republic of China	5	none
Australia	5	none
Canada	4	University of Toronto (1038), The University of British Columbia (566)
Japan	3	none
The Netherlands	3	none
Denmark	2	none
Belgium	2	none
Switzerland	2	none
Germany	2	none
Sweden	2	none
Singapore	1	none
Republic of Korea	1	none
Hong Kong	1	none
Finland	1	none

### 3.3. Institutions with highest numbers of authors

Table S2 in the supplementary material and table 2 present information on the 116 institutions that have more than 10 000 authors (and up to 35 547) with five or more full publications. These institutions have between 606 and 5027 authors eligible for the primary analysis, with the lowest value observed at Pfizer, Inc.—apparently few authors with pharma affiliation publish many papers. The number of top-cited authors varies vastly, from 14 (Southern Medical University) to 1894 (Harvard University) for all top-cited authors and from 14 to 1135 in the primary analysis. The proportion of top-cited authors in the primary analysis also showed wide variation from 1.05% (Capital Medical University) to 40.13% (UC Berkeley). Several institutions in this list of the largest institutions have very low proportions of top-cited scientists. In 13 institutions, this proportion is below 6% in the primary analysis, including 8 institutions in People's Republic of China and others in Russia, India, Mexico, Czech Republic and Brazil.

**Table 2.** Number of authors in the 116 institutions with over 10 000 authors. Data per region, with details shown on top 20 institutions. Detailed data for all 116 institutions appear in table S2 in the supplementary material.

region	among the 116	among top 20
USA	38	Harvard University (26366), Stanford University (24041), University of Michigan, Ann Arbor (22335), Johns Hopkins University (21268), University of Pennsylvania (20953), University of California, Los Angeles (20358), University of Washington (19683)
People's Republic of China	27	Shanghai Jiao Tong University (35547), Zhejiang University (31288), Peking University (27753), Tsinghua University (24487), Huazhong University of Science and Technology (24022), University of Chinese Academy of Sciences (23373), Sichuan University (23245), Fudan University (21490), Central South University (20139), Jilin University (18882)
France	8	none
UK	7	none
Germany	4	none
Japan	4	The University of Tokyo (18748)
Canada	4	University of Toronto (30477)
Switzerland	2	none
Sweden	2	none
Belgium	2	none
Italy	2	none
Australia	2	none
Republic of Korea	2	none
The Netherlands	2	none
Taiwan	1	none
Finland	1	none
Norway	1	none
Czechia	1	none
Russia	1	none
Singapore	1	none
India	1	none
Denmark	1	none
Brazil	1	Universidade de São Paulo (23723)
Mexico	1	none

### 3.4. Institutions with highest percentage of top-cited authors

Table S3 in the supplementary material and table 3 show the 100 institutions with the highest proportions of top-cited authors (range, 30.43–79.17%) among all 6979 institutions. The top 20 of tables 1 and 3 have no institutions in common. Most of the table 3 institutions are small- or modest-size organizations, and many of them are research institutes and companies in the tech space. The top 100 include only 30 universities and polytechnics (supplementary material, table S3).

**Table 3.** Percentage of top-cited authors (career-long impact) in institutions with the highest percentage of top-cited authors in the primary analysis. Data per region, with details shown on top 20 institutions. Detailed data for all 100 institutions appear in table S3 in the supplementary material.

region	number among top 100	among top 20
USA	38	Meta FAIR (69.23%), Institute for Advanced Study (64.71%), National Bureau of Economic Research (57.14%), California Institute for Quantitative Biosciences (50.00%), Santa Fe Institute (50.00%), Howard Hughes Medical Institute (42.86%), Princeton University (41.93%), National Center for Health Statistics (41.86%)
Germany	13	Max-Planck-Institut für Eisenforschung GmbH (47.62%), CISPA - Helmholtz Center for Information Security (46.15%), Max Planck Institute for Coal Research (43.33%), Max Planck Institute for Informatics (42.11%)
UK	13	The Medical Research Council Laboratory of Molecular Biology (47.17%)
Spain	8	CSIC - Instituto de Ciencia y Tecnologia de Alimentos y Nutricion (ICTAN) (40.38%)
The Netherlands	3	Wolters Kluwer (79.17%)
Canada	3	Perimeter Institute for Theoretical Physics (58.62%)
Taiwan	2	none
France	2	INSEAD, Europe (56.25%), EURECOM (42.42%)
Switzerland	2	none
Italy	2	none
Hong Kong	2	none
Türkiye	2	none
Ukraine	1	none
People's Republic of China	1	none
Kuwait	1	none
Palestine, State of	1	none
Norway	1	none
Iraq	1	none
Iran, Islamic Republic of	1	none
India	1	National Aerospace Laboratories India (42.11%)
United Arab Emirates	1	Mohamed Bin Zayed University of Artificial Intelligence (44.00%)
Austria	1	none

For institutions with a small number of authors in the primary analysis, their prominently high placement may be spurious or occasionally may even point to gaming practices, since a few gamed top-cited authors can propel a small institution to high placement. To avoid challenges with small numbers of eligible authors, table S4 in the supplementary material and table 4 show the top 100 institutions with the highest proportion (24.84–41.93%) of top-cited authors in the primary analysis, when limited to the 2380 institutions with at least 100 authors eligible for the primary analysis. The top 20 in tables 1 and 4 have little overlap.

**Table 4.** Percentage of top-cited authors (career-long impact) in institutions with the highest percentage of top-cited authors in the primary analysis limited among the institutions with at least 100 authors for the primary analysis. Data per region, with details shown on top 20 institutions. Detailed data for all 100 institutions appear in table S4 in the supplementary material.

region	number among top 100	among top 20
USA	62	Princeton University (41.93%), University of California, Berkeley (40.13%), Carnegie Mellon University (38.22%), Alphabet Inc. (37.29%), University of California, Santa Barbara (36.38%), Massachusetts Institute of Technology (34.16%), Rockefeller University (31.93%), Georgia Institute of Technology (31.77%), Rice University (31.36%), University of Maryland, College Park (30.91%), University of Illinois Urbana-Champaign (30.22%), Microsoft Corporation (30.20%) and The University of Texas at Austin (30.11%)
UK	17	The James Hutton Institute (33.98%), London School of Economics and Political Science (32.41%), University of Cambridge (31.86%) and University of Oxford (30.66%)
Hong Kong	5	Hong Kong University of Science and Technology (31.83%) and The Education University of Hong Kong (30.53%)
Canada	3	none
Israel	3	none
Switzerland	2	ETH Zürich (32.37%)
People's Republic of China	1	none
The Netherlands	1	none
Türkiye	1	none
Ireland	1	none
Spain	1	none
Singapore	1	none
Germany	1	none
Saudi Arabia	1	none

### 3.5. Retractions, self-citations and discontinued titles

The publicly deposited files provide information for each institution on retraction authorships ΣR across all authors who had at least five full published papers and started publishing in 1980 or later, top-cited authors in the primary analysis with high self-citations *S(t,p),* and top-cited authors in the primary analysis with high rates of items published in discontinued titles *D(t,p).* Many institutions have high values when contrasted to the number of top-cited authors and to all eligible authors in the primary analysis, suggesting that their ranking will be markedly affected when these parameters are also considered.

Prevalence of institutions among the adjustment components shows large differences by country. When we divide each component-specific penalty by the number of top-cited primary analysis authors *N(t)* for each institution, we get the relative impact of the adjustments of each component on the institution. For retractions, the top 100 institutions ranked by retraction adjustment per *N(t)* are heavily concentrated in People's Republic of China (75/100 institutions). These 75 People's Republic of China institutions with high retractions account for 18% of all Chinese institutions among the 2380. Other countries with at least two institutions in the top 100 of retractions were Indonesia (*n* = 3, 19% of its total institutions), Malaysia (*n* = 2, 10%), Morocco (*n* = 2, 25%) and Republic of Korea (*n* = 2, 3%). For self-citation, the top 100 institutions ranked by the self-citation adjustment per *N(t)* are dominated by Russia (*n* = 26, 52% of its total institutions), People's Republic of China (*n* = 9, 2%), Indonesia (*n* = 7, 44%), Poland (*n* = 6, 15%) and the Czech Republic (*n* = 6, 35%). For discontinued titles, the top 100 institutions ranked by the discontinued-title adjustment per *N(t)* are led by India (*n* = 12, 13%), People's Republic of China (*n* = 12, 3%), Malaysia (*n* = 9, 45%), Saudi Arabia (*n* = 6, 32%) and Russia (*n* = 4, 8%).

### 3.6. Percentile ranking after adjustment for research integrity indicators

Table S5 in the supplementary material and table 5 show the top 100 institutions according to the percentile ranking that corrects for retractions, self-citations and discontinuations when limited to the 2380 institutions with at least 100 authors who started publishing in 1980 or later, have published 40 or more Scopus-indexed items in their career, and these include at least five single, first- or last-authored items. For the 2380 institutions, their percentile ranking is available in the publicly deposited files. As shown, the adjusted percentile ranking for many institutions is markedly different from the unadjusted percentile ranking using only the proportion of top-cited authors. The Spearman correlation between the penalties incurred for each of the three research integrity components is –0.05 for retractions and self-citations, 0.22 for retractions and discontinued titles and 0.12 for self-citations and discontinued titles when all 2380 institutions are considered.

**Table 5.** Corrected percentage of top-cited authors (career-long impact) in institutions with the highest percentage of top-cited authors after correction for three research integrity proxies, in the primary analysis limited among the institutions with at least 100 authors for the primary analysis. Data per region, with details shown on top 20 institutions. Detailed data for all 100 institutions appear in table S5 in the supplementary material.

region	number among top 100	among top 20
USA	62	Alphabet Inc. (40.20%), Carnegie Mellon University (37.73%), University of California, Berkeley (37.26%), Princeton University (36.47%), Massachusetts Institute of Technology (32.97%), Microsoft Corporation (32.61%), University of California, Santa Barbara (32.24%), Georgia Institute of Technology (31.35%), Woods Hole Oceanographic Institution (31.03%), National Institute of Diabetes and Digestive and Kidney Diseases (NIDDK) (30.11%), Rockefeller University (30.08%), Rice University (29.74%), Colorado School of Mines (28.65%) and University of Illinois Urbana-Champaign (27.59%)
UK	15	The James Hutton Institute (36.41%), University of Cambridge (28.10%) and University of Oxford (27.61%)
Canada	4	none
Hong Kong	4	Hong Kong University of Science and Technology (31.08%)
Switzerland	2	ETH Zürich (31.05%) and École Polytechnique Fédérale de Lausanne (29.19%)
Israel	2	none
The Netherlands	2	none
Ireland	1	none
Türkiye	1	none
France	1	none
Italy	1	none
Spain	1	none
Japan	1	none
Sweden	1	none
Denmark	1	none
Germany	1	none

As shown in table 6, People's Republic of China has the largest number of institutions (*n* = 410) among the 2380 compared with any other country, followed by the USA (*n* = 344), while Japan, France and UK also have more than 100 institutions each in that list. A total of 28 countries/regions have at least 15 institutions among the 2380. UK, The Netherlands, Switzerland, Canada, USA, Australia and Sweden have very high percentile rankings of their institutions with very similar median percentile values (82.6th–85.5th percentile). Conversely, Saudi Arabia, People's Republic of China, Malaysia, Iran, India and Indonesia have very low percentile rankings of their institutions, with median values ranging from 3.9th to 21st percentile. These countries are penalized the most from the three adjustments, with the median cumulative adjustment exceeding their number of top-cited authors *N(t)* by 1.29–2.75-fold.

**Table 6.** Regions with at least 15 institutions among the 2380 institutions with > = 100 primary-analysis authors: counts, median adjusted percentile and median cumulative adjustment (as proportion of *N(t)*). Detailed data for all 2380 institutions appear in the supplementary excel file sheet raw data.

region	number of institutions among the 2380	median percentile ranking among the 2380 (using adjusted baseline)	median cumulative adjustment (as proportion of *N(t)*)
People's Republic of China	410	14.3	2.06
USA	344	83.6	0.13
Japan	152	43.0	0.39
France	137	58.5	0.18
UK	117	85.5	0.10
Italy	96	50.8	0.34
Germany	95	74.8	0.18
India	92	18.8	1.29
Spain	80	50.1	0.21
Republic of Korea	71	36.7	0.62
Russia	50	27.9	0.82
Brazil	47	33.5	0.53
Australia	46	83.2	0.13
Türkiye	46	33.1	0.73
Canada	42	84.0	0.09
Poland	41	43.0	0.38
Iran, Islamic Republic of	34	15.4	1.70
Taiwan	32	56.6	0.40
Malaysia	20	15.3	1.61
Saudi Arabia	19	3.9	2.75
The Netherlands	18	84.5	0.08
Switzerland	17	84.5	0.14
Czechia	17	38.3	0.60
Romania	17	26.6	0.90
Finland	16	73.8	0.09
Indonesia	16	21.0	2.01
Sweden	15	82.6	0.12
Austria	15	71.5	0.22

### 3.7. Single recent-year analyses and sensitivity analyses

Analyses using the single recent-year data and sensitivity analyses are presented in Appendix Results.

## 4. Discussion

We provide data that allow standardized assessment of institutions worldwide based on the number of publishing authors with each specific institutional affiliation, the concentration of top-cited authors in each institution and three indicators that may offer proxies of diverse research integrity aspects (retractions and high self-citation and publication at discontinued title rates).

The percentage of top-cited authors can be calculated for different sets of authors. Here, we preferred for the primary analysis a set that considers only authors whose publication activity spans a typical research-career window and who have a substantial publication record and at least five published items with leading and/or senior authorship positions. Such data allow the use of a standardized process for calibrating institutional impact through the proportion of its top-cited authors. Defining the set of authors based on objective publication age and productivity criteria regardless of what their academic or other rank and status creates some common ground for comparisons of very diverse institutions. Academic positions, ranks and status would be extremely difficult to standardize across thousands of diverse institutions. These institutions include not only universities (that already have different systems for classifying faculty and staff) but also many other organizations and entities that publish scientific papers.

Science-wide mapping of nearly 7000 institutions reveals that top-cited scientists are disproportionately concentrated in high numbers in a few institutions. However, there are many other institutions of more modest size or even relatively small size that have high proportions of top-cited authors among their ranks. Without adjusting by size, the excellent profiles of these institutions would have been buried under the volume of the largest ones and remained unnoticed. Institutions with high values include not only large universities but also research institutes of more modest size and several tech companies and other organizations. Conversely, among the very largest institutions (with size determined by their numbers of total authors), there is extremely large variability as to the presence and percentage of top-cited scientists.

Our databases may be used for research assessments of institutions. However, we caution that, in principle, institutional rankings may be problematic as extensively discussed before [1–4,23–25]. Frustrated with the misuse of institutional rankings, some initiatives such as COARA have called even for their total avoidance [4]. Instead of total abolition, a more pragmatic operationalization is to use ranking metrics more judiciously [26], generate more standardized metrics [27] and consider also systematic appraisals of gaming processes [28,29] to better accommodate research integrity. The three proxy indicators of research integrity may best be seen as offering warning signals rather than validated proof of research integrity failure at an institutional level. The same cautious principles apply also to interpreting the summary score ranking which should be seen as heuristic rather than some gold standard of validated ranking. If our data are used for any assessment and ranking purposes, they should be strictly interpreted for what they show. Clearly, they may not capture sufficiently many other dimensions that are important for understanding the impact of research and other organizations, e.g. education, policy, community work, health services and more.

Our analysis also reveals that focus on top-cited authors may be one-sided and may not account for aspects that touch on research integrity. We demonstrate that some institutions (mostly small ones) from countries with very limited resources have extremely high percentages of top-cited authors. This pattern should be interpreted with caution, and similar patterns have been linked in prior literature to concerns about publication integrity challenges [30–32]. We used here also retraction data to penalize the citation impact of institutions against the volume of authors with retracted papers where the retraction has not been owing to publisher/journal error. We also used percentile-adjusted information on rates of self-citations and publications in discontinued titles to further calibrate the profile of each institution. Therefore, the generated summary score considers both impact and aspects of research integrity.

Some institutions may have high concentrations of top-cited authors while also showing signals that warrant closer integrity-related examination. Other recent analyses by *Nature* [33] have also revealed that some institutions, especially in People's Republic of China, Saudi Arabia, India, Pakistan and Ethiopia are indeed retraction hotspots. Accounting for retractions, inordinate self-citations and publications in discontinued titles can substantially alter the placement of some institutions in the adjusted rankings. We hope that inspection of our data may thus strengthen proper incentives, weaken the drive for publication and citation misconduct and identify institutions and author groups that require more in-depth scrutiny. Different countries may have macro-environments that generate different types of problems. For example, inspection of the list of institutions with the highest penalties shows that many Chinese institutions are penalized for high retractions, while many Russian institutions are penalized for high self-citations and high percentage of publications in discontinued titles. Overall, in our baseline analysis, Saudi Arabia, People's Republic of China, Malaysia, Iran, India and Indonesia have cumulative penalties that on average exceed their number of top-cited authors. However, as already discussed, none of the three proxies offer full proof of research integrity problems at the institution level, let alone the country level. While they can serve as warning signals, alternative explanations may also exist why their values are high in specific countries. Country-level interpretations should be tempered by the realization that findings may be affected by factors beyond research integrity, including disciplinary composition, publication growth, collaboration patterns, database coverage, institutional age and even bias that may lower retraction barriers for authors from some specific countries. Moreover, discipline-level differences are prevalent both in retraction rates and in self-citations and discontinued titles. Percentile ranks are relative to the selected sample of 2380 institutions and do not represent an absolute measure of institutional or national integrity.

There are several limitations in our work. First, while the precision and recall of Scopus are high, some authors may be misclassified regarding top-cited status because of inaccuracies. Second, assigning a single affiliation to each author also carries uncertainty and the potential for error. Institutions interested in more in-depth assessments of their people may thus wish to verify data against their up-to-date personnel files. For small institutions, we caution that even small errors can cause substantial changes in the number and proportion of highly cited authors. Therefore, while we make available the full data on 6979 institutions, only the largest one-third of them may be immune to the impact of even very modest errors. Uncertainty for each studied proportion can be estimated by standard 95% confidence intervals for proportions, e.g. using Wilson score interval method especially for small samples. For example, for Wolters Kluwer, 19/24 primary analyses authors being top-cited corresponds to a proportion of 79% with a relatively wide 95% confidence interval of 59–90%, while confidence intervals for large institutions are very tight, e.g. for Harvard University 1135/4191 is 27.08% (25.76 to 28.45%). Third, we have used an aggregation system for organizations and there can be disagreements on specific aggregations. Requests for re-evaluation and revision of aggregation decisions should be sent directly to Scopus, as we do not personally have permission to make any changes in the Scopus system ourselves. Notably, some institutions currently are under pressure to merge [34,35]. While there may be some sensible reasons for such mergers, e.g. reduction of redundancy in resources or cost, other reasons include misaligned incentives—in particular, gaining higher ranks in university rankings that depend on volume and size. Fourth, retracted papers are probably only a small fraction of the fraudulent and highly flawed papers that should be retracted [36]. We cannot exclude that the fraction is different across different institutions. However, it is impossible to estimate that fraction. Therefore, use of the observed retractions is the best available option. Fifth, some retractions may be owing to honest error [37], and scientists recognizing their errors and correcting the record should be applauded. However, retraction notices are usually too elliptical to separate securely honest error from misconduct, and the latter is probably the large majority [38]. Finally, while we provide theoretical justification for our adjustment and correction approach to generate a summary score, eventually the exact weighting of the adjustments and corrections is a largely arbitrary choice. Within a reasonable sensitivity range, different corrections for self-citations, discontinued titles and retractions do not materially alter the overall institutional ordering, but a subset of institutions can still change substantially. Moreover, if science-wide data becomes available for other proxies of research integrity, these may also be considered in the future for additional multi-dimensional institutional assessments.

Our approach of combining positive and negative impact indicators, with the latter informed by research integrity metrics, can be considered in the context of other initiatives. The RI², introduced in 2025, combines field-normalized indicators concerning retractions, publication in discontinued journals, self-citation and citations to retracted research into an institutional composite score and corresponding risk tiers [15]. Our penalty scheme therefore partially overlaps with the indicators used in RI². SCImago's IRIS platform, launched in 2026, applies closely related indicators—institutional self-citation, discontinued-journal output and retractions—alongside multi-affiliation, hyperauthored output, leadership impact gap, hyperprolific authors, institutional journal output and redundant output to produce a normalized institutional score relative to peers [39]. The precise construction and normalization of the indicators differ across these initiatives, and neither combines positive and negative impact indicators. Moreover, our approach draws evidence at the author level, with particular attention to top-cited authors, since these are major drivers of an institution's or even a country's impact. Nevertheless, our approach and these two initiatives share the objective of drawing systematic attention to integrity-related signals and may serve as early-warning tools for institutions.

Finally, our assessment relies strongly on top-cited authors and their ranks may sometimes be reached by scientists who do not necessarily have genuine high impact but engage in spurious hyperprolific behaviour [14,40]. However, the composite indicator that we use for assigning the top-cited status does not count at all the number of publications and is resistant to hyperprolific behaviour, unless this is for papers as single, first or last author. Empirical probes suggest that only few of the listed top-cited authors exhibit overtly problematic patterns for various reasons [41].

Overall, the data that we make publicly available may help improve transparency for institution-level impact and research integrity issues among the authors who are currently affiliated with each institution. Careful balance of excellence and research integrity is needed.

## Supplementary Material



## Data Availability

The key data are available in Elsevier Data Repository [42]. Supplementary material is available online [43].
