# Supplementary material for: Science-wide mapping of institutions based on affiliated authors' impact and research integrity proxies
Source: R Soc Open Sci. 2026 Oct 14;13(10):260870. doi: 10.1098/rsos.260870 (PMC13682425; doi:10.1098/rsos.260870)
Supplement: Supplementary file 1 [file rsos.260870.zip › rsos.260870/rsos260870_si_001.docx]

**ELECTRONIC SUPPLEMENTARY MATERIAL**

**Science-wide mapping of institutions based on affiliated authors’ impact and research integrity proxies**

John P.A. Ioannidis (1*), Jeroen Baas (2), Roy Boverhof (2), Cyril Voyant (3*)

Royal Society Open Science doi: 10.1098/rsos.260870

**SUPPLEMENTARY TABLES AND FIGURES**

All supplementary tables and figures have been posted at Data Mendeley at doi: 10.17632/jn5j7gjpzj.1

**SUPPLEMENTARY TEXT**

**APPENDIX METHODS**

**Aggregation of affiliations**

Scopus displays a single organization to each Scopus author ID. For authors who have multiple organizations in their published corpus, only one organization is selected based on an automated algorithmic procedure that prioritizes the last listed organization in the most recent papers. Authors can also curate their data and select their own preferred organization based on the list of recent organizations. Authors belonging to the same institution may nevertheless use different ways to present their organization in their work. This may result in heterogeneity and dispersal of the authors of the same institution across different organizations. For example, a scientist affiliated with Harvard, may be shown with their organization being the university (“Harvard University”, n=3699 authors), the school of the university (e.g. “Harvard Medical School” n=15,016, or “Harvard T.H. Chan School of Public Health” n=1,872, or “Harvard Business School” n=283) or occasionally even more circumscribed entities and units within the university (e.g., “The Rowland Institute at Harvard” n=47, or “Smithsonian Astrophysical Laboratory” n=68).

For this project, we use these granular organizations, as reported in the Scopus Author ID for each author to generate “aggregated” organizations which we refer to as institutions. For the institutions all underlying (child) organizations that pertain to the same institution are combined, with the exception that organizations that name only hospitals are kept separate. For example, the institution for Harvard University aggregates 50 different organizations that have at least one author assigned to them among the 10,933,183 authors. However, it does not aggregate hospitals that may have Harvard affiliation. Organizations such as “Massachusetts General Hospital”, “Massachusetts General Hospital Cancer Center”, and “MGH Institute of Health Professions” are merged under a separate institution named Massachusetts General Hospital. A small number of organizations may be assigned to multiple different institutions; these are referred to as “jointly owned” organizations.

The full hierarchy of organizations that are mapped to an institution upon aggregation can be found in the subscription edition of Scopus on the organization profile page by navigating to the “structure” tab. The inclusion and exclusion criteria of these organizations are based on the relationship between the parent organizations and their child-organizations in the hierarchy. Relationships between organizations can be attributable or non-attributable. In the case of an attributable relationship, all output of the child-organization is rolled up to the parent organization and the child-organization is visible in the institutional hierarchy on Scopus. This tree-view of an organizational hierarchy can contain multiple levels of organizations, in other words child-organizations may also have their own child-organizations.

Organizations are more granular than the institutions authors are linked to as these largely reflect the choice of how each author represents themselves (which organization they have listed on their publications). The institutional aggregation achieves far better standardization in this regard, but is also not perfect, e.g. some authors with “Massachusetts General Hospital” listed organizations may have appointments at Harvard University, like those who get aggregated under “Harvard University”, and vice versa.

**APPENDIX RESULTS**

**Single recent-year analyses**

Analyses using the single recent-year instead of career-long data provided qualitatively similar inferences (see Supplementary Tables 6-10). Numbers and proportions of top-cited authors were on average slightly higher, given that the list of top-cited authors includes more authors in the recent-year list than in the career-long list. The increase was disproportionately higher for some institutions, especially some of the largest and most high-impact ones (e.g. Harvard and Stanford), and furthermore most prominent for Chinese universities. Focusing on single recent-year impact propels several small institutions from non-high-income countries to the top ranks of proportion of top-cited authors (Supplementary Table 8). However, almost all these institutions are markedly affected by authors with retracted papers and high self-citation and discontinuation rates suggesting gaming of metrics and misconduct in their environment. Focusing on institutions with at least 100 eligible authors for the primary analysis (Supplementary Table 9) removes the very small spurious institutions, but several mid-size institutions predominantly from Saudi Arabia and some from Egypt, India, and United Arab Emirates appear at the very top ranks in the absence of adjustments, but almost all of them lose their top-ranks or even end up with negative adjusted numbers. This may reflect gaming practices (as captured by retractions and high self-citations) or the common use of Saudi Arabian institutional affiliations by scientists in other countries who are paid heavy bonuses to do this. The Spearman correlation between the penalties incurred for each of the 3 research integrity components is -0.12 for retractions and self-citations, 0.16 for retractions and discontinued titles, and 0.11 for self-citations and discontinued titles when all 2380 institutions are considered.

**Sensitivity analyses**

Sensitivity analyses excluding from the primary analysis authors who had not actively published since 2020 removed about 12% of authors from the analysis but yielded qualitatively similar inferences (not shown).

Across all sensitivity analyses on different p and f adjustments, institutional percentile rankings remained highly correlated with the baseline specification (Supplementary Figures S1-8). As shown in Supplementary Figure S1 for the career-long impact, Spearman correlations ranged from 0.928 to 0.991 across all combinations of percentile cutoffs (p = 80, 95, 99) and retraction-penalty factors (f = 1, 2, 4) with the baseline of p=95 and f=2. Bland-Altman analyses (Supplementary Figure S2) confirm that most institutions deviate only modestly from baseline, although a small minority demonstrate shifts of approximately 20–40 percentile points under the most extreme correction settings. Scatterplots of adjusted percentages (Supplementary Figure S3) show equally strong agreement, and KDE plots (Supplementary Figure S4) demonstrate that the overall distribution of adjusted institutional scores changes only minimally under alternative parameter settings.

Sensitivity analyses for the single-year citation impact produced qualitatively similar patterns (Supplementary Figures S5-8) but with slightly lower correlations and somewhat greater dispersion (Spearman correlations ranging from 0.897 to 0.986). These sensitivity analyses support the use of the baseline specification (p = 95, f = 2) as a pragmatic reference choice, as results remain largely consistent across a wide range of alternative parameter settings.
